# Real life use of dolutegravir doravirine dual regimen in experienced elderly PLWH with multiple comorbidities and on polypharmacy: A retrospective analysis

**DOI:** 10.1097/MD.0000000000028488

**Published:** 2021-12-30

**Authors:** Maria Mazzitelli, Lolita Sasset, Davide Leoni, Cristina Putaggio, Anna Maria Cattelan

**Affiliations:** a Infectious and Tropical Diseases Unit, University Hospital of Padua, Padua, Italy; b Magna Graecia University of Catanzaro, Catanzaro, Italy.

**Keywords:** dolutegravir, doravirine, dual therapy, HIV, PLWH, real-life

## Abstract

By increasing life expectancy of people living with HIV, the most clinical challenge is managing both drug-to-drug interactions and comorbidities (especially metabolic). Doravirine (DOR), a new non-nucleoside reverse transcriptase inhibitor, recently approved for the treatment of HIV, could be a good companion of dolutegravir (DTG) in a dual regimen for experienced elderly patients with multimorbidity and polypharmacy.

We herein report our preliminary experience in a small cohort of elderly patients (>50 years of age) with multimorbidity and on polypharmacy who were switched to DOR/DTG dual regimen and followed-up for 3 months. The study was conducted at the Infectious and Tropical Diseases Unit of Padua University Hospital, Italy.

Eighteen patients were included, 72.2% males and 27.8% postmenopausal women, mean age was of 61.3 years (7.6), 50% experienced AIDS events. Switches to DOR and DTG were mainly due to high cardiovascular and metabolic risk (72.2%), and interactions among comedications (50%). Antiretrovirals that subjects were switched off were mostly boosted protease inhibitors 66.7%. We observed a viral suppression among all subjects. Interestingly, we observed a statistically significant reduction in body mass index, body weight and waist circumference, eGFR, and a significant increase in serum creatinine levels. No significant changes in CD4+ T cell count was observed from the baseline. Lipid and fasting glucose values did not change significantly.

To the best of our knowledge this is the first experience reporting real-life outcome of switch to DTG + DOR in elderly with multimorbidity and on polypharmacy. From our very preliminary data the dual combination of DTG and DOR could be a good treatment strategy for these subjects. However, our findings need to be validated on a greater number of patients.

## 1 Introduction

Doravirine (DOR) is a new non-nucleoside reverse transcriptase inhibitor recently approved for the treatment of HIV-1 infection. It exists both as single agent and in combination with tenofovir diproxil emifumarate. European AIDS Clinical Society guidelines recommend the combination of DOR with tenofovir diproxil emifumarate as first line agent in naïve patients, while American guidelines prefer to limit its use in specific sub-groups of patients.[^1^,^2^]

Results from clinical trials confirmed a good safety profile of DOR both on HIV virological control and on metabolic profile.[^3^,^4^,^5^] Moreover, when compared with other agents of the same class, DOR seems to have a better tolerability profile, less interactions with food or drugs, and a higher genetic barrier.^[6]^

Prevalence of polypharmacy ranges from 37% to 94% in elderly people living with HIV (PLWH), and increases by age, as a direct consequence of an increasing proportion of multimorbidity in this population.^[7]^ Therefore, managing PLWH with multimorbidity and polypharmacy is to date a significant challenge for clinicians, and requires implementation of dedicated multidimensional clinics.^[8]^ Our objectives were to describe and report our preliminary clinical experience with the combination of DOR and dolutegravir (DTG) in a small group of experienced elderly PLWH with multiple comorbidities and in polypharmacy and to assess clinical and laboratory impact of this combinations (viro-immunological parameters – HIV RNA and CD4+ T cell count, body weight, lipids, glucose, and kidney function).

## 2 Methods

This study was conducted at Infectious and Tropical Diseases Unit of University Hospital of Padua and received approval form the Hospital Board. Patients’ consenting was waived as per Italian law for retrospective studies (Italian Drug Agency note, March 20, 2008, no. 76). Data of patients treated with DOR and DTG for 3 months (June–August 2021) were retrospectively collected from clinical health records. Multimorbidity and polypharmacy were defined as per literature.[^9^,^10^] Student *t* test and Mann Whitney *U* test were used to compare continuous variables (normally and not-normally distributed, respectively). Chi squared test was used to assess categorical variables, as appropriate. Level of significance was set up with a *P* value < .05.

## 3 Results

**Figure 1:**
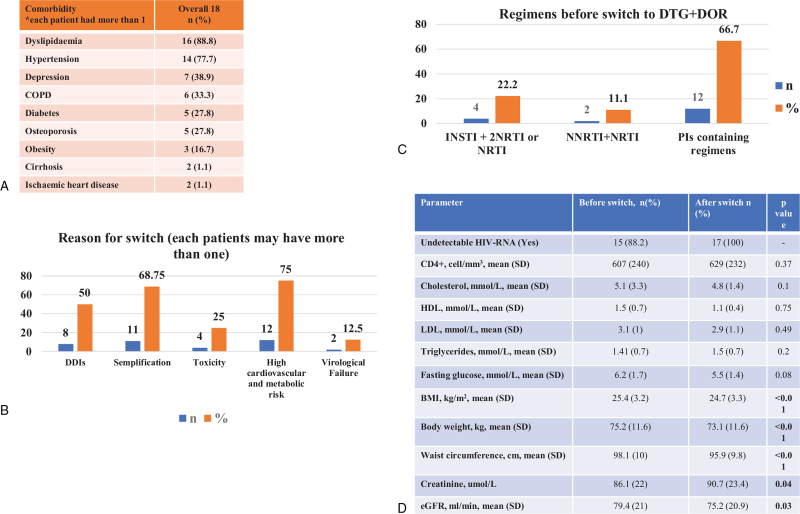
Summary of the study results.

Eighteen patients were included in this analysis, 13 (72.2%) males and 5 (27.8%) postmenopausal women. Mean age was of 61.3 years (standard deviation: 7.6), with a mean length of HIV disease of 23 years (standard deviation: 8.5). Nine patients (50%) experienced AIDS events in their past medical history. Risk factors for HIV acquisition were mainly intravenous drug use (8/18, 44.4%) and unprotected sex with men (6/18, 33.3%). All patients had multimorbidity and polypharmacy, with severe polypharmacy in 5/18 (27.8%) cases. Comorbidities and class of comedications are depicted in Figure 1, panel A. Most frequent comorbidities were dyslipidaemia (93.7%), hypertension (77.7%), and depression (38.9%). Fifteen subjects (83.3%) had 2 or more reasons to have their previous art regimen changed. Switches to DOR and DTG were mainly due to high cardiovascular and metabolic risk (13/18, 72.2%), and interactions among comedications (9/18, 50%) (Fig. 1, panel B). Interactions among medications were due in all cases to anti-psychiatric agents [interacting with boosted protease inhibitors (PIs)] and in 1 case to protonic-pump inhibitor (interacting with rilpivirine). Antiretrovirals that subjects were switched off were mostly boosted protease inhibitors, PIs in 66.7% (Fig. 1, panel C). In Figure 1, panel D, are depicted main lab and metabolic interest clinical values before and after switch, for 17 patients, because 1 patient stopped DOR and DTG regimen for psychiatric side effects before the follow-up analysis. No other patients experienced side effects. A man reported *Clostridium difficile* diarrhoea for 3 days, due to an ongoing antibiotic treatment. As for viro-immunological parameters, we observed a maintenance in viral suppression among all those who were undetectable at the baseline. Two people who were not undetectable at the baseline showed undetectable HIV RNA during the follow-up. Interestingly, we observed a statistically significant reduction (*P* < .05) in body mass index, body weight and waist circumference, eGFR, and a significant increase in serum creatinine levels (Fig. 1, panel D). No significant changes in CD4+ T cell count, lipids and fasting glucose were observed from the baseline.

## 4 Discussion

To the best of our knowledge, even if very limited by the low numbers, this is one of the first real-file experiences describing the impact of DTG + DOR dual regimen. Fear of possible worsening of comorbidities (especially cardiovascular disease) in patients who were on a boosted PI was the main driver for switch in our small cohort, as well as the drug interactions perpetrated by the boosting agents. The major issue, in the era in which the burden of antiretroviral drugs decreased with the introduction of dual regimen, is the increasing number of non-cART medications. Psychiatric comedications are one of the most involved class in drug interactions, especially when administered with PIs.

The effect on body weight and decrease of waist circumference, even if significant, need to be confirmed in greater cohort and now not conclusive. It could be more likely explained by the remotion of PIs from the antiretroviral regimen more than a “slimming effect” of DOR and we are not still able to declare if it will be maintained over time, and whether the presence in the regimen of DTG will contribute to weight gain. No significant effect was observed on lipids and glucose levels, probably because people with dyslipidaemia were on statin and people with diabetes on antidiabetic drugs daily, respectively, but also this finding will require future confirmation. The effect on kidney function could be explained by the decreased tubular secretion of creatinine induced by DTG (which was not present in the baseline regimen), rather than a real renal toxicity of the studied combination.^[11]^ However, all these results are very preliminary and need to be interpreted with caution until more data will be available (especially on a greater number of patients, with prospective studies and with a longer follow-up time).

From our very preliminary data the dual combination of DTG and DOR seems to be an option for elderly subjects, with multiple comorbidities and drug interaction/toxicity issues, and can be a patient's tailored treatment, even if not currently contemplated as association by guidelines. Further data on greater number of PLWH and with longer follow-up are necessary to confirm our results.

## Author contributions

MM and AMC were involved in designing the study. LS, DL and CP collected the data. MM analyzed the data and drafted the manuscript. All authors (MM, AMC, LS, DL) were involved in revising the article.

**Conceptualization:** Maria Mazzitelli, Anna Maria Cattelan.

**Data curation:** Maria Mazzitelli, Lolita Sasset, Davide Leoni, Cristina Putaggio, Anna Maria Cattelan.

**Formal analysis:** Maria Mazzitelli.

**Methodology:** Maria Mazzitelli.

**Project administration:** Maria Mazzitelli.

**Supervision:** Maria Mazzitelli, Anna Maria Cattelan.

**Writing – original draft:** Maria Mazzitelli, Anna Maria Cattelan.

**Writing – review & editing:** Maria Mazzitelli, Lolita Sasset, Davide Leoni, Cristina Putaggio, Anna Maria Cattelan.
